# Effects of Fermented Mushroom of *Cordyceps sinensis*, Rich in Selenium, on Uterine Cervix Cancer

**DOI:** 10.1155/2014/173180

**Published:** 2014-05-25

**Authors:** Jing Ji, Juan Liu, Haijuan Liu, Yueling Wang

**Affiliations:** ^1^Department of Gynecology and Obstetrics, First Affiliated Hospital of Medical School, Xi'an Jiaotong University, Xi'an 710061, China; ^2^Department of Obstetrics, Maternal & Child Health Care Hospital of Shanxi Province, Xi'an 710003, China

## Abstract

The purpose of this study was to investigate the effect of fermented mushroom of * Cordyceps sinensis *(CS), rich in selenium (Se-CS), on uterine cervical cancer in mice. The methylcholanthrene- (MCA-) induced tumor model was used in this paper. After the mice were administered Se-CS, the animals showed 40% tumor incidence (*P* < 0.05). Se-CS also enhanced the immune functions. Se-CS treatment showed significant (*P* < 0.05–0.01) restoration in the level of glutathione content, lipid peroxidation, glutathione peroxidase activity, glutathione reductase activity, catalase activity, Na^+^/K^+^-ATPase activity, and glutathione S transferase activity. This finding suggested that the concomitant use of Se and CS could be a potential therapeutic approach to improve the efficacy of therapy for uterine cervical cancer.

## 1. Introduction

Uterine cervical cancer is still the second most common cancer in women worldwide, despite the existence of effective screening methods [1, 2]. However, the treatment causes strong side effects such as digestive symptoms (vomiting and diarrhea) and bone marrow suppression. Thus, drugs with fewer side effects and a superior effect in combination are desired.

In humans, selenium (Se) is a trace element nutrient which functions as cofactor for reduction of antioxidant enzymes such as glutathione peroxidases. Several studies have suggested a possible link between cancer and selenium deficiency [3–5]. Some reports show that selenium administered to laboratory animals at levels above dietary requirements is capable of protecting against tumor formation in the mammary glands, liver, skin, colon, stomach, oral cavity, bladder, and pancreas [6–8]. However, it is toxic if taken in excess. Exceeding the tolerable upper intake level of 400 mg per day can lead to selenosis [9]. The present study evaluated the optimal concentration of Se, at which Se has equal efficacy but much lower toxicity. One novel selenium complex of Se-CS has been designed and evaluated. Using trace element at lower doses, in combination with edible mushrooms, has been ascribed as one potent way to reduce trace elements-associated toxicity and maintain their effect [10].

*Cordyceps sinensis *(CS) is a popular medicinal mushroom that has been used as a home remedy in traditional Chinese medicine for the prevention or treatment of a variety of diseases including cancer [11, 12]. Today, CS is recognized as a dietary supplement recommended in many countries as a cancer therapeutic. However, some of the trails were not well designed and lacked appropriate controls [2]. In the current study, we investigated the potential therapeutic efficacy of Se-CS for cervical cancer. The purpose of this study was to investigate the effect of fermented mushroom of Se-CS on methylcholanthrene-induced uterine cervical cancer in mice.

## 2. Materials and Methods

### 2.1. Animals

This study was performed in accordance with the Guide for the Care and Use of Laboratory Animals. Care was taken to minimize discomfort, distress, and pain to the animals.

Female Kunming strain mice were maintained at room temperature under alternating natural light/dark photoperiod and had access to standard laboratory food and fresh water* ad libitum.*

### 2.2. Sodium Selenite Solution (SS)

Sodium selenite was dissolved in saline solution (0.9% NaCl). An ampule was filled with 0.4 mL of SS and then was sterilized in a microwave oven for 3 minutes.

### 2.3. Se-Enriched* Cordyceps sinensis *(Se-CS)

Se-CS was prepared according to study of Zhang et al. [9]. The only difference was that a series of 0 mg, 0.1 mg, 0.2 mg, 0.4 mg, 0.6 mg, 0.8 mg, and 1 mg sodium selenite solutions was added to the substrate for obtaining Se-CS samples 1 (Se-0), 2 (Se-0.1), 3 (Se-0.2), 4 (Se-0.4), 5 (Se-0.6), 6 (Se-0.8), and 7 (Se-1), respectively [13, 14].

### 2.4. The Effect of Se on Growth of CS

The mycelia were harvested at the end of fermentation; then they were centrifuged at 12,000 g for 10 min and dried to constant weight at 60°C for sufficient time in laboratory vacuum ovens, and the dry weight of the mycelia was then measured.

### 2.5. Analysis of Organic Selenium in Se-CS

Se-CS was dialyzed against distilled water for 96 h by changing the water every 12 h until no Se was detected in the dialyzing water. Thus, Se compounds left in the dialyzed sample were considered to be organic selenium. The Se content was determined using graphite furnace atomic absorption spectrophotometry introduced by Zhang et al. [9]. Briefly, 100 grams of properly homogenized, dry mycelia was placed in a vitreosil crucible overnight at 410°C–440°C in an electric muffle furnace, maintaining the temperature 1200°C. The dry-ashing method destroys all of the organic materials present in the sample. The crucible containing pure ash was then taken out of the muffle furnace and kept in a desiccator [9]. Two grams of ash was digested with a mixture of hydrochloric acid and nitric acid in the ratio 1 : 3 [9]. The digested sample was dissolved in 50 mL of distilled water and used for analysis by means of an atomic absorption spectrometer (AAS-3200, Shanghai, China). The wavelength on selenium analyses was 196.0 nm.

Se concentrations in liver and kidney were determined using atomic absorption spectrometer (AAS-3200, Shanghai, China). The standard of selenium was purchased from Anhui Star New Material Technology Co., Ltd, China. Weighed aliquots of frozen tissue were digested in 3 stages: the first using 5 mL mixed acids (4 : 1 nitric acid : perchloric acid), the second using a combination of 2 mL HNO_3_ and 30% H_2_O_2_, and finally using 2 mL HNO_3_. All digestion stages were performed at 130°C until the acid was completely evaporated and the residue dried before the next acid stage was started. After the third acid treatment, 1% HNO_3_ was added to the digests and heated at 80°C for 1 h. After cooling, the sample volume was measured and analyzed.

### 2.6. Tumor Induction by MCA

Murphy's string method [15] was followed for the induction of tumors in the uterine cervix of mice. Briefly, sterile double cotton thread impregnated with beeswax containing 600 *μ*g of MCA was inserted into the canal of the uterine cervix by means of laparotomy under mild ether anaesthesia. Forty-eight of these mice were allocated equally into 4 groups: MCA-induced group (Group 1), MCA and Se-0.4 group (Group 2), MCA and SS group (Group 3), and MCA and Se-0 group (Group 4). The other 12 normal mice were used as the control group (Group 5). From then on, the 5 groups of mice were administered orally saline, Se-CS-0.4, SS-0.4, Se-CS-0, and saline, respectively. Body weight of the animals was recorded initially, at fortnightly intervals and at autopsy. All animals surviving after 90 days were killed. Tumor incidences in control and experimental groups were calculated [16]. Tissue samples of thymus, spleen, liver, and kidney were dissected from the visceral tissues. After washing with saline, the tissue samples were blotted dry and weighed. The uterus tissues of the mice were dissected for the estimation of various parameters related to oxidative stress. All samples were stored at −80°C for future analysis.

### 2.7. The Impact of Se-CS on Immune Organ

The impact of Se-CS on immune organ was evaluated based on the thymus index and spleen index [17]. The thymus or spleen index was calculated by the following formula: thymus (spleen) index = weight of thymus (spleen) (mg)/weight of mouse (g).

### 2.8. Biochemical Estimations

In uterus tissues, lactate dehydrogenase (LDH) was estimated using a method described by Lum and Gambino [18]. Serum was used for the assay of glutathione (GSH) content, lipid peroxidation (LPO), glutathione peroxidase (GPx) activity, glutathione reductase (GR) activity, catalase (CAT) activity, Na^+^/K^+^-ATPase activity, and glutathione S transferase (GST) activity by enzyme linked immunosorbent assay (ELISA) using DSL-10-1600 Active ELISA kit (Shanghai Jinma Biological Technology, Inc., China) [19–24].

### 2.9. Data Analysis

All data were analyzed by a one-way analysis of variance, and the differences between means were established by Duncan's multiple-range test. The data represents means and standard deviations. The significant level of 5% (*P* < 0.05) was used as the minimum acceptable probability for the difference between the means.

## 3. Results

### 3.1. The Effect of Se on Growth of CS

**Figure 1:**
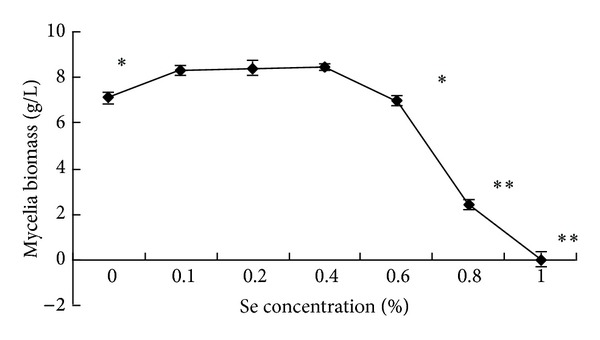
The effect of Se on growth of* Cordyceps sinensis.* The biomass of mycelia was the maximum when there was 0.4% Se in the medium. It declined rapidly when the concentration of Se exceeded 0.6%. ^∗^ *P* < 0.05 and ^∗∗^ *P* < 0.01 versus 0 mg Se.

Se can be accumulated by CS even from low external concentrations (Figure 1). But it is also known that Se interpolates into disulfide bridges of protein, causing a structural weakness that leads to selenosis [25]. The biomass of mycelia was significantly influenced by the concentration of Se in the medium (Figure 1). A lower concentration of Se stimulated noticeably the growth of mycelium as compared to medium without selenium. But it was no significant variety as the concentration of Se between 0.1–0.4%. However, at 0.6% of Se the growth was clearly inhibited (*P* < 0.05).

### 3.2. Organic Selenium in Se-CS

**Figure 2:**
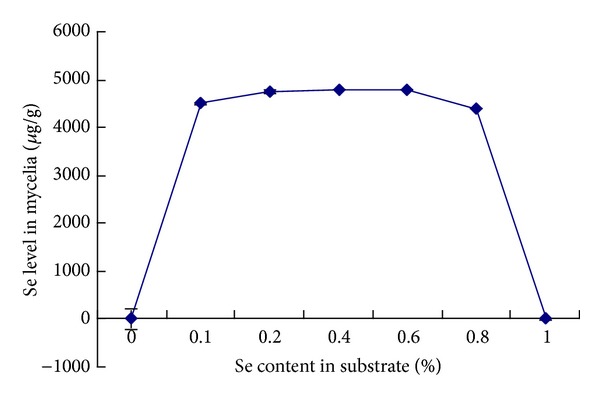
The content of Se in the mycelia. The content of Se accumulated in the mycelia was 4789.26 ± 13.0 *μ*g/g, when the concentration of Se in the medium was 0.4%. *n* = 6. Values are means ± SEM.

The determination of selenium in mycelia obtained in the absence and in the presence of Se in medium demonstrated the incorporation of element to fungal cells (Figure 2). Apparently, there was no significant increase of selenium level in mycelia grown in 0.6% Se as compared to 0.4% Se (*P* > 0.05). Thus, we chose 0.4% Se as the optimal concentration of Se added in the liquid culture.

### 3.3. Effect of Se-CS on Survival of Mice Treated with MCA

The mice did not suffer from any apparent toxic effect of Se-0.4 during the observation period. Only a small number of mice died in certain groups (Table 1).

### 3.4. The Effect of Se-CS on Tumor Incidence

MCA-induced group administered orally saline showed 85.7% tumor incidence, whereas MCA and Se-0.4 group showed 40% tumor incidence (*P* < 0.05). SS-treated group and Se-0-treated group showed 77.7% and 70% tumor incidence, respectively. Although tumor incidence in SS-treated group and Se-0-treated group was lower than that of MCA-induced group, the difference was not significant (*P* > 0.05). Animals of control group showed no cervical tumor incidence (Table 2).

### 3.5. The Effect of Se-CS on Immune Function

As shown in Table 3, compared with the Se-CS treated group, the thymus index and spleen index of mice in the MCA-treated group decreased significantly (*P* < 0.05). However, there was no significant difference between the MCA-treated group and the SS-treated group (Table 3). These results indicated that Se-CS could enhance the immune functions.

### 3.6. Se Accumulation in Liver and Kidney 

The organ masses of liver and kidney were significantly different between SS and Se-CS group (*P* < 0.05). However, there was no significant difference of organ masses of liver and kidney between the Se-0.4 and Se-0 groups (*P* > 0.05). It is implied that SS-0.4 is toxic to animals, while Se-CS is essentially nontoxic (Table 4).

**Figure 3:**
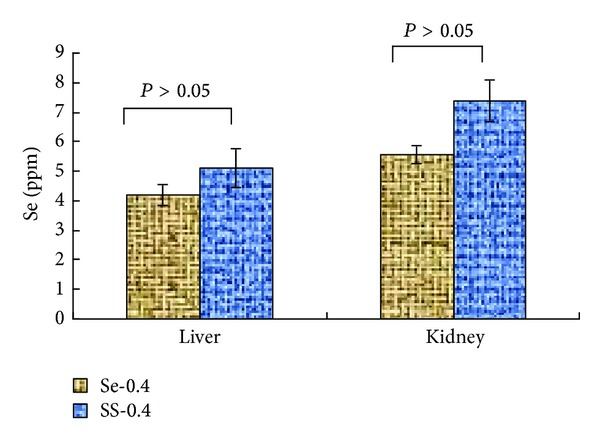
Se (ppm) in animal tissues (*n* = 6). The Se (ppm) was not significantly different between SS-0.4 and Se-0.4 group (*P* > 0.05).

The Se contents in animal tissues were measured following the studies and the results are shown in Figure 3. The Se content in tissues of Se-0.4 group was 4.21 ppm and 5.56 ppm (liver and kidney) while SS-0.4 groups exhibited elevated levels of Se content but not to a statistically significant degree (*P* > 0.05). It is implied that CS could reduce Se accumulation in animal tissues to a certain extent.

### 3.7. The Effect of Se-CS on Various Parameters Related to Oxidative Stress

LDH was measured to evaluate the role of antioxidative stress in the protection of Se-CS. A significant increase in the activity of LDH in serum was observed in MCA group, as compared to the control group (*P* < 0.01), whereas Se-CS treatment significantly (*P* < 0.05) resulted in decreased serum LDH levels when compared with MCA group mice (Table 5). Concentrations of GSH were lower in MCA group than those in control group (Table 5). Se-CS produced the increase in the level of GSH (*P* < 0.05). The level of LPO content adds to the proof of the increased peroxidative damage during MCA. A significant increase (*P* < 0.001) in the content of LPO was observed in the MCA group when compared with the control group. In the Se-CS group, a significant decrease (*P* < 0.05) was seen in the level of LPO when compared with the MCA group (Table 5).

Activities of various antioxidant enzymes and Na^+^/K^+^-ATPase of different groups have been listed in Table 6. The activity of endogenous antioxidant enzymes was decreased significantly (*P* < 0.01) in the MCA group, as compared to the control group, whereas, in the Se-CS group, Se-CS treatment showed significant (*P* < 0.05–0.01) restoration in the level of various enzymes as compared with MCA group.

## 4. Discussion

In the current study, we investigated the potential therapeutic efficacy of Se-CS for cervical cancer. Se is required in biosynthesis of important selenoenzymes. Some of them are active as catalysts for reduction of extracellular oxidants, thereby protecting cells from potential damage by these hazardous compounds. CS is recognized as a dietary supplement recommended in many countries as a cancer therapeutic. So the coeffect of CS and selenium on uterine cervix cancer was studied.

During the production of Se-CS, the biomass of mycelia was significantly influenced by the Se in the medium (Figure 1). This result is consistent with the findings of another study by Han et al. [13]. The possible mechanism could be that vanadium influences the stability of cell membranes, as well as the syntheses of nucleic acids and the stability of the double helix of DNA while forming hydrogen bonds.

Survival of animals receiving Se-CS was significantly longer than the groups receiving only CS or Se. The shortest survival times were observed in the no treatment group. The findings of the present study demonstrate that oral administration of Se-CS during the process of MCA-induced uterine cervix cancer results in significant reduction in the occurrence of cervical carcinomas (*P* < 0.05–0.01). CS could enhance the immune functions and reduce Se accumulation in animal tissues to a certain extent. The two agents achieve cytotoxicity through different means. It is possible that the bifunctional modulator of them inhibits tumor induction.

However, Se is also known to be toxic with a narrow range separating chronic conditions of deficiency and toxicity. The molecular toxicity of inorganic selenium was described in relation to its interaction with endogenous –SH groups. The animal studies have demonstrated that liver and kidney are the major target organs of Se toxicity [26]. The Se contents in animal tissues were measured following the studies. Ingestion of Se with CS reduced tissue metal accumulation, particularly for liver and kidney. At the same time, the organ masses of liver and kidney were significantly different between SS and Se-CS group (*P* < 0.05). The results indicate that Se-CS is less toxic to mice than SS.

Previous reports have shown that immunosuppression can be clearly detected in both cancer patients and tumor-bearing animals, demonstrating that the immune system plays an important role in immunosurveillance against malignant cells [27]. Many attempts have been made during the past years to develop immunostimulating approaches to cancer treatment. Thymus and spleen are important immunological organs that indirectly reflect humoral immunity. Some kinds of immune inhibitors could cause thymus and spleen atrophy. Our study demonstrated that spleen and thymus indexes were significantly decreased in MCA-treated group mice. This is consistent with the conclusion that immune system plays an important role in immunosurveillance against malignant cells [27]. However, the decrease of thymus and spleen indexes caused by MCA treatment in mice was alleviated by Se-CS. It demonstrated that improved immune function achieved by Se-CS may translate into an antitumor effect. Future clinical trials are required to confirm this finding.

It has been proposed that antioxidant changes reflect an altered redox balance in several pathological states [28]. LDH was measured to evaluate the role of antioxidative stress in the protection of Se-CS. It resulted in decreased serum LDH levels when compared with MCA group mice. GSH is one of the primary endogenous antioxidant defense systems, which removes hydrogen peroxide and lipid peroxides. Decline in GSH levels could either increase or reflect oxidative status [29, 30]. Se-CS produced the increase in the level of GSH (*P* < 0.05). The large numbers of polyunsaturated fatty acids make cell membranes particularly vulnerable to lipid peroxidation. The oxidation of polyunsaturated fatty acids alters the structure of the membrane with resultant changes in fluidity and permeability. LPO can also inhibit the function of membrane bound receptors and enzymes [22]. In the Se-CS group, a significant decrease was seen in the level of LPO when compared with the MCA group. The antioxidants would be consumed in the reaction with free radicals. Therefore, the measurement of endogenous antioxidants enzymes, that is, GPx, GR, CAT, and GST, as well as Na^+^/K^+^-ATPase, has been performed to estimate the amount of oxidative stress. Se-CS treatment showed a significant (*P* < 0.05–0.01) restoration in the level of various enzymes as compared with MCA group. These results suggested that Se-CS on uterine cervical cancer might be directly through a lower extent of oxidative stress in MCA mice.

## 5. Conclusions

Collectively, our results indicate that Se-CS may represent a novel protective strategy against uterine cervical cancer by attenuating oxidative stress and improving immune function in MCA mice. However, the antitumor effect of Se-CS-0 and SS was not significant. It is implied that the antitumor effect was caused by the coeffect of CS and Se. Furthermore, our results also emphasize that Se-CS is less toxic to mice than SS. This finding suggested that the concomitant use of Se and CS could be a potential therapeutic approach to improve the efficacy of therapy for uterine cervical cancer.

## Figures and Tables

**Table 1:** Effect of SE-CS and other treatments on survival of MCA-treated mice.

Different groups	Number of mice	
	Initial	Final
MCA-treated	12	7
MCA and Se-0.4-treated	12	10
MCA and Se-0-treated	12	10
MCA and SS-treated	12	9
Control group	12	11

**Table 2:** Effect of SE-CS and other treatments on tumor incidence induction by MCA.

Different groups	Number of final mice (*n*)	Number of mice with cervical squamous cell carcinoma (*n*)	Tumor incidence (%)
MCA-treated	7	6	85.7
MCA and Se-0.4-treated	10	4	40*
MCA and Se-0-treated	10	7	70
MCA and SS-treated	9	7	77.7
Control group	11	0	0

*P < 0.05 versus MCA-treated group.

**Table 3:** The effect of SE-CS on immune function (*n* = 7–11).

Different groups	Number of mice (*n*)	Thymus index (×10^3^)	Spleen index (×10^3^)
MCA-treated	7	1.73 ± 0.980	5.17 ± 0.56
MCA and Se-0.4-treated	10	2.78 ± 0.06*	7.51 ± 0.10*
MCA and Se-0-treated	10	2.56 ± 0.03*	7.35 ± 0.23*
MCA and SS-treated	9	2.06 ± 0.09	5.94 ± 0.30
Control group	11	2.67 ± 0.23*	8.04 ± 0.29*

*P < 0.05 versus MCA-treated group.

**Table 4:** Effects of Se-CS on organ masses of mice (*n* = 7–11).

Mouse group	Number of mice (*n*)	Liver weight (g)	Kidney weight (g)
Control group	7	1.47 ± 0.05^a^	0.49 ± 0.04^a^
MCA-induced group	10	1.30 ± 0.07^b^	0.37 ± 0.03^b^
MCA and SS group	10	1.31 ± 0.06^b^	0.34 ± 0.03^b^
MCA and Se-0 group	9	1.48 ± 0.07^a^	0.48 ± 0.04^a^
MCA and Se-0.4 group	11	1.45 ± 0.06^a^	0.46 ± 0.02^a^

The different letters in the same column indicate a statistical difference (*P* < 0.05).

**Table 5:** Effect of SE-CS and other treatments on serum LDH, GSH, and LPO levels.

Different groups (*n*)	LDH (IU/L)	GSH (nmol/mg protein)	nmol/g protein
Control (7)	85.222 ± 2.561**	1.39 ± 0.003**	13.21 ± 0.26**
MCA-induced group (10)	181.111 ± 3.630	0.56 ± 0.021	22.20 ± 1.01
MCA and SS group (10)	170.121 ± 1.220	0.61 ± 0.011	22.11 ± 2.01
MCA and Se-0 group (9)	162.100 ± 2.130	0.79 ± 0.031	20.22 ± 3.22
MCA and Se-0.4 group (11)	132.222 ± 2.201*	1.16 ± 0.055*	16.88 ± 0.11*

Values are shown as means ± SEM. *P < 0.05 versus MCA group; **P < 0.01 versus MCA group.

**Table 6:** Effect of SE-CS and other treatments on the activity of various enzymes (nmol/g protein).

Different groups (*n*)	GPx	GR	GST	CAT	Na^+^K^+^-ATPase
Control (7)	16.08 ± 1.13***	36.50 ± 3.51***	18.01 ± 1.12**	7.88 ± 0.13*	4.52 ± 0.32*
MCA-induced group (10)	7.01 ± 0.32	20.89 ± 2.01	9.07 ± 1.11	3.20 ± 0.13	2.00 ± 0.13
MCA and SS group (10)	7.31 ± 0.30	21.86 ± 1.11	9.87 ± 2.21	3.88 ± 0.83	2.20 ± 0.11
MCA and Se-0 group (9)	7.98 ± 0.22	23.13 ± 2.31	10.07 ± 3.22	4.00 ± 0.83	3.00 ± 0.10
MCA and Se-0.4 group (11)	10.10 ± 0.32*	25.01 ± 2.12***	11.60 ± 0.88*	4.78 ± 0.23*	3.23 ± 0.11*

Values are shown as means ± SEM. *P < 0.05 versus MCA group, **P < 0.01 versus MCA group, and ***P < 0.001 versus MCA group.
